# Overexpression of Galectin 3 in Pancreatic β Cells Amplifies β-Cell Apoptosis and Islet Inflammation in Type-2 Diabetes in Mice

**DOI:** 10.3389/fendo.2020.00030

**Published:** 2020-02-07

**Authors:** Ivica Petrovic, Nada Pejnovic, Biljana Ljujic, Sladjana Pavlovic, Marina Miletic Kovacevic, Ilija Jeftic, Aleksandar Djukic, Nevena Draginic, Marijana Andjic, Nebojsa Arsenijevic, Miodrag L. Lukic, Nemanja Jovicic

**Affiliations:** ^1^Center for Molecular Medicine and Stem Cell Research, Faculty of Medical Sciences, University of Kragujevac, Kragujevac, Serbia; ^2^Department of Pathophysiology, Faculty of Medical Sciences, University of Kragujevac, Kragujevac, Serbia; ^3^Department of Genetics, Faculty of Medical Sciences, University of Kragujevac, Kragujevac, Serbia; ^4^Department of Histology and Embryology, Faculty of Medical Sciences, University of Kragujevac, Kragujevac, Serbia; ^5^Department of Pharmacy, Faculty of Medical Sciences, University of Kragujevac, Kragujevac, Serbia

**Keywords:** galectin 3, type-2 diabetes, β cells, apoptosis, inflammation

## Abstract

**Aims/Hypothesis:** Galectin 3 appears to play a proinflammatory role in several inflammatory and autoimmune diseases. Also, there is evidence that galectin 3 plays a role in both type-1 and type-2 diabetes. During obesity, hematopoietic cell-derived galectin 3 induces insulin resistance. While the role of galectin 3 expressed in islet-invading immune cells in both type-1 and type-2 diabetes has been studied, the importance of the expression of this molecule on the target pancreatic β cells has not been defined.

**Methods:** To clarify the role of galectin 3 expression in β cells during obesity-induced diabetogenesis, we developed transgenic mice selectively overexpressing galectin 3 in β cells and tested their susceptibility to obesity-induced type-2 diabetes. Obesity was induced with a 16-week high-fat diet regime. Pancreatic β cells were tested for susceptibility to apoptosis induced by non-esterified fatty acids and cytokines as well as parameters of oxidative stress.

**Results:** Our results demonstrated that overexpression of galectin 3 increases β-cell apoptosis in HFD conditions and increases the percentage of proinflammatory F4/80^+^ macrophages in islets that express galectin 3 and TLR4. In isolated islets, we have shown that galectin 3 overexpression increases cytokine and palmitate-triggered β-cell apoptosis and also increases NO_2−_-induced oxidative stress of β cells. Also, in pancreatic lymph nodes, macrophages were shifted toward a proinflammatory TNF-α-producing phenotype.

**Conclusions/Interpretation:** By complementary *in vivo* and *in vitro* approaches, we have shown that galectin 3-overexpression facilitates β-cell damage, enhances cytokine and palmitate-triggered β-cell apoptosis, and increases NO_2−_-induced oxidative stress in β cells. Further, the results suggest that increased expression of galectin 3 in the pancreatic β cells affects the metabolism of glucose and glycoregulation in mice on a high-fat diet, affecting both fasting glycemic values and glycemia after glucose loading.

## Introduction

Diabetes mellitus is a group of heterogeneous metabolic disorders that is characterized by chronic hyperglycemia caused by defect in insulin secretion due to pancreatic β-cell damage, defects in its effect, or both (1–3). Both type-1 and type-2 diabetes lead to functional and structural damage to β cells. This may be induced by an autoimmune process in type-1 diabetes or, in type 2, due to high glycemia and/or lipid levels. Type-2 diabetes is linked to obesity and genetic predisposition, where insulin secretion fails to compensate for insulin resistance. Obesity-associated insulin resistance is a key characteristic of type-2 diabetes and metabolic syndrome and develops as a result of ongoing inflammation in obese white adipose tissue (4). Chronic exposure of the β cell to elevated fatty acid concentrations leads to decreased glucose-induced insulin secretion, impaired insulin gene expression, and increased cell death (5). Evidence suggests that endoplasmic reticulum (ER) stress contributes to β-cell loss and insulin resistance. ER stress may also contribute to cytokine-induced β-cell death (6). During the development of type-2 diabetes, chronic hyperglycemia leads to a vicious cycle of continuous deterioration of β-cell function (5).

High-fat diet (HFD)-induced metabolic dysfunction in mice is a useful model for studying the pathogenesis of type-2 diabetes (7). Increased caloric intake leads to the development of obesity (8). In obesity, white adipose tissue becomes resistant to the antilipolytic effect of insulin, and the concentration of non-esterified fatty acids (NEFA) increases. As a consequence, the fat-binding capacity of fat cells reduces, and NEFA cause a further rise in insulin resistance in muscle, liver, and pancreatic β cells, accompanied by impaired insulin secretion (9). During obesity and diabetes, adipose tissue and pancreatic islets become infiltrated by macrophages and undergo inflammation that further impairs insulin signaling. Macrophage-mediated inflammation participates in β-cell dysfunction by local release of cytokines in obese or type-2 diabetes states. It is also indicated that the metabolic stress induced by hyperglycemia and high NEFA levels can trigger increased cytokine and chemokine production from β cells, causing increased blood monocyte recruitment with an accumulation of M1-like islet macrophages (10).

Galectin 3 belongs to the family of lectins, evolutionarily conserved proteins with various roles. Galectin 3 is an ~30-kDa protein and, by its structure, is unique among members of the family of galectins (11). It contains one carbohydrate-binding site (CRD) region coupled to a non-lectin amino-terminal chain of about 120 amino acid lengths with short repeating sequences rich in proline and glycine. In the presence of multivalent ligands, the galectin-3 molecules merge into pentamers and in this way cross-link the surface glycoconjugates by forming a lattice structure (12–14). It is primarily present in epithelial and endothelial cells of various tissues and organs and sensory neurons, as well as in the cells of the immune system, monocytes and macrophages, dendritic cells, neutrophils, eosinophils, basophils, and mast cells (13, 14). Extracellular galectin 3 mediates adhesion and activation of cells (15) and may have proapoptotic activity (16). Galectin 3 appears to be a proinflammatory molecule in several inflammatory and autoimmune diseases (17). There is also evidence that galectin 3 plays a role in both type-1 and type-2 diabetes. In type-1 diabetes, expression of galectin 3 on accessory cells, in particular dendritic cells, appears to lead to activation of inflammatory lymphocytes and macrophages and is required for β-cell damage (18).

While the role of galectin 3 in invading immune cells has been studied, the importance of the expression of this molecule on the target β cells *in vivo* is not defined. *In vitro* data have provided conflicting evidence. Early studies indicated that IL-1β-stimulated rat islets upregulated galectin 3, which protected β cells against IL-1β toxicity (19). On the other hand, a deficiency of galectin 3 due to genetic deletion or application of chemical inhibitor protects pancreatic islets from TNF-α^+^IFN-γ^+^IL-1β-triggered apoptosis (20).

In this study, we aimed to investigate the role of the galectin 3 expressed in β cells in HFD-induced metabolic defects. To clarify the role of galectin 3 expression in β cells during obesity-induced diabetogenesis, we used transgenic mice selectively overexpressing galectin 3 in β cells. We show that overexpression of galectin 3 promotes β-cell apoptosis in HFD conditions and increases the percentage of proinflammatory F4/80^+^ macrophages in islets that express galectin 3 and Toll-like receptor 4 (TLR4). Further, we present data that galectin 3 overexpression increases cytokine and palmitate-triggered β-cell apoptosis and NO_2−_ induced oxidative stress in β cells. Thus, in complementary *in vivo* and *in vitro* approaches, we show that galectin 3 overexpression facilitates β-cell damage, enhances cytokine, and palmitate-triggered β-cell apoptosis, and increases NO_2−_-induced oxidative stress in β cells.

## Materials and Methods

### Experimental Mice and Study Design

We used wild-type C57BL/6J male mice (WT) and littermate C57BL/6J mice with transgenically enhanced galectin 3 expression in the pancreatic β cells (TG), 8–10 weeks old, obtained in collaboration with Prof. Bernard Thorens (Center for Integrative Genomics, University of Lausanne). To generate transgenic mice expressing galectin 3 in pancreatic islet β-cells, the galectin 3 cDNA was subcloned in front of the rat insulin promoter, as previously described (21). Transgenic mice in C57Bl/6 background were prepared by a commercial service. For testing the mouse genotype, we extracted DNA from ear tissue (KAPA Express Extract, KK7102, Kapabiosystems, USA). For PCR reaction, we used the KAPA 2G Fast Ready Mix PCR Kit (KK5102, Kapabiosystems, USA) and the primers listed in Supplementary Material. Overexpression of galectin 3 in the pancreatic β cells was confirmed with 591 bp PCR product visualized on agarose gel (Supplement 1). All experimental animals were bred in our animal facilities under standard laboratory conditions in a temperature-controlled environment with a 12 h light/dark cycle and received water and a standard low-fat diet (LFD, 10% calories from fat, Mucedola, Italy) or a high-fat diet (HFD, 60% calories from fat, Mucedola, Italy) *ad libitum* for 16 weeks. We used 15–20 animals per group in two repeated experiments. Mice were sacrificed by cervical dislocation, and blood samples, visceral adipose tissue, and pancreas were collected for further analyses.

### Ethics Statement

The study was conducted in compliance with all the regulations and recommendations stated in the European Union Directive 2010/63/EU. All animal procedures were approved by the Ethical Committee of the Faculty of Medical Sciences, University of Kragujevac (Permit Number: 01-6675).

### Metabolic Parameters

The body weight of mice and glycemic events were monitored at regular intervals. Mice were fasted for 4 h, and glucose levels (mmol/L) were measured using an Accu-Chek Performa Glucometer (Roche Diagnostics, Mannheim, Germany). Fasting insulin levels in serum were measured using the Mouse Insulin ELISA Kit (CSB E05071m, Cusabio biotech). Homeostatic Model Assessment of Insulin Resistance (HOMA-IR) was quantified as blood glucose level multiplied by serum insulin level divided by 22.5. Soluble galectin 3 levels in serum were measured using the galectin 3 mouse ELISA Kit (ab203369, Abcam). After sacrifice, the amount of the epididymal visceral adipose tissue (VAT) was measured for each mouse. Serum concentrations of lipids were measured using the Olympus AU600 Chemistry Immuno Analyzer (Olympus, Tokyo, Japan).

### Intraperitoneal Glucose Tolerance Test

An intraperitoneal glucose tolerance test (ipGTT) was performed after 16 weeks of HFD feeding. Mice were fasted for 12 h prior to the test. At baseline, the blood glucose level was measured. Glucose was administered at Time 0 (1.5 g/kg per mouse, Sigma-Aldrich, Germany), and then blood glucose levels were recorded at 15, 30, 90, 60, 90, and 120 min. Animals were fully conscious throughout the ipGTT.

### Histopathological Analyses

Pancreata were excised and placed in 10% buffered formaldehyde fixative solution for 24 h at room temperature. Paraffin-embedded pancreata sections (5 μm) were stained with hematoxylin-eosin and were used for the analysis of mononuclear infiltrates in the Langerhans pancreatic islets by light microscope (BX51; Olympus, Japan) using a 40X magnification lens. Histological analysis of the distribution of inflammatory cell infiltrate in pancreatic islets was performed in a blinded fashion by two independent observers. Insulitis was graded, and a mean insulitis score was calculated as described previously (22). For every specimen, we made at least five serial sections of whole pancreas of ~200 μm depth. A minimum of 50 islets/group were scored for insulitis under double-blinded conditions. The degree of insulitis was graded according to the following: normal islet, score 1; perivascular/periductal infiltration, score 2; peri-insulitis, score 3; mild insulitis (<25% of the islet infiltrated), score 4; and severe insulitis (more than 25% of the islet infiltrated), score 5.

### Immunohistochemistry

For immunohistochemical staining, we used paraffin-embedded sections (5 μm) of mouse pancreas tissue. Deparaffinized tissue sections were incubated with primary mouse anti TLR-4 antibody (ab22048, Abcam), Galectin 3 antibody (ab53082, Abcam), anti-CD68 antibody (ab49777, Abcam), or Insulin antibody (ab63820, Abcam). Staining was visualized by using the mouse-specific HRP/DAB detection IHC kit (ab64259, Abcam), and sections were counterstained with Mayer's hematoxylin. Sections were photomicrographed with a digital camera mounted on a light microscope (Olympus BX51, Japan), digitized, and analyzed. Quantification was performed using ImageJ software (National Institutes of Health, Bethesda, MD, USA; http://rsb.info.nih.gov/ij/) on non-overlapping 10 fields/section. Scoring and histological analysis were performed in a blinded fashion by two independent observers. The islet area was calculated using the software, and then the immunoreactive area of the islet was calculated using a threshold on the red color channel. We did not investigate the intensity of the immunopositive staining since it is not applicable to DAB chromogen. Results are presented as a mean count of positive cells per field or percentage of islet area.

### Isolation and Cultivation of Primary Mouse Islets of Langerhans

Pancreatic islets were isolated from TG and WT mice by the collagenase V digestion technique, followed by handpicking (23). Before performing experiments, islets were cultured overnight in RPMI-1640 medium containing 10% fetal calf serum (FCS, PAA Chemicals, Pasching, Austria), 10 mM HEPES, 5 mM 2-mercaptoethanol, 2 mM L-glutamine, 1 mM sodium pyruvate, 100 IU/ml penicillin, and 100 mg/ ml streptomycin (culture medium) in a humidified (5% CO, 95% air) atmosphere at 37°C. After recovery, islets were incubated in culture medium in 24-well non-adhesive culture plates or in 96-well flat-bottomed tissue culture plates. For apoptosis analysis, a cell suspension was made using Gibco™ Cell Dissociation Buffer (Gibco, Fisher Scientific Ltd., Finland). Cells were stained using an Annexin V Apoptosis Kit [FITC] (Novus Biological, United Kingdom) according to the manufacturer's protocol and analyzed using a FACS Calibur (BD Biosciences) and FlowJo software (Tree Star). The analysis strategy is shown in Supplement 2.

### Treatment of Pancreatic Islets With Palmitic Acid and Cytokines

All reagents were from Sigma (St. Louis, MO, USA) and all dishes for culturing cells from Sarstedt (Numbrecht, Germany), unless stated differently. To make the stock solution for further dilution in RPMI-1640, palmitic acid (PA) was mixed overnight at 37°C in Krebs Ringer HEPES buffer containing 20% BSA (fraction V, Roche, Basel, Switzerland). Groups of 20 islets were subjected to *in vitro* tests. Islets were treated with PA (100 μmol/l) for 24 h. Islets were also treated for 24 h with a proinflammatory cytokine cocktail. The following recombinant cytokines were used in the experiments: rat IFN-γ (R&D Systems, MI, USA, 10 ng/ml), mouse IL-1β (R&D Systems, MI, USA, 10 ng/ml), and mouse TNF-α (R&D Systems, MI, USA, 10 ng/ml).

### Isolation of Pancreatic Lymph Node Cells for Phenotypic Assessment and Flow Cytometry Analysis

Pancreatic draining lymph nodes were removed, and single-cell suspensions were obtained by mechanical disruption. After removing pancreatic lymph nodes, pancreas was processed through three steps: *in-situ* perfusion with collagenase, pancreatic digestion, and isolation of the islets. The cells were separated according to the protocol described elsewhere (23) and analyzed by flow cytofluorimetry.

Islet cells were stained with either combinations of fluorochrome-labeled primary antibodies or isotype controls for 30 min at 4°C. For intracellular staining, cells were activated with PMA/ionomycin and processed as previously described (24). Cells were stained using an Annexin V Apoptosis Kit [FITC] (Novus Biological, United Kingdom) according to the manufacturer's protocol and analyzed using a FACS Calibur (BD Biosciences) and FlowJo software (Tree Star). The analysis strategy is shown in Supplement 2. Cells were labeled with fluorochrome-conjugated monoclonal antibodies: anti-mouse CD45 (553079, BD Biosciences), F4/80 (MF48020, Invitrogen), TLR4 (12-9041-80, eBioscience), Galectin 3 (12-5301-82, eBioscience), GLUT-2 (FAB1440A, R & D Systems, Minneapolis, MN), CD3 (553067, BD Biosciences), CD11c (MCD11C05, Invitrogen), CD206 (ABTU0111121, R & D Systems, Minneapolis, MN), and TNF-α (130-102-294, MACS Miltenyi Biotec). The cells were analyzed using a FACS Calibur (BD Biosciences) and FlowJo software (Tree Star).

### Biochemical Assays

Oxidative stress parameters were determined spectrophotometrically (Specord S-600 Analytik Jena) using collected samples of pancreas islet supernatant. The level of the superoxide anion radical (O_2−_) was measured via a nitro blue tetrazolium reaction in TRIS buffer at 530 nm. Krebs-Henseleit solution was used as a blank probe (25). The measurement of the level of hydrogen peroxide (H_2_O_2_) was based on the oxidation of phenol red by hydrogen peroxide in a reaction catalyzed by horseradish peroxidase (HRPO) (38). A volume of 200 μL of perfusate was precipitated using 800 mL of freshly prepared phenol red solution; 10 μL of (1:20) HRPO (made ex tempore) was subsequently added. For the blank probe, an adequate volume of Krebs Henseleit solution was used. The level of H_2_O_2_ was measured at 610 nm (26). Nitric oxide decomposes rapidly to form stable nitrite/nitrate products. The nitrite level (NO_2−_) was measured and used as an index of nitric oxide (NO) production using Griess's reagent. A total of 0.5 mL of supernatant was precipitated with 200 μL of 30% sulpho-salicylic acid, vortexed for 30 min and centrifuged at 3,000 g. Equal volumes of the supernatant and Griess's reagent, containing 1% sulphanilamide in 5% phosphoric acid/0.1% naphthalene ethylenediamine-di hydrochloride were added and incubated for 10 min in the dark and measured at 543 nm. The nitrite levels were calculated using sodium nitrite as the standard (27). The degree of lipid peroxidation in the islet cell supernatant was estimated by measuring thiobarbituric acid reactive substances (TBARS), using 1% thiobarbituric acid in 0.05 NaOH, which was incubated at 100 C for 15 min and measured at 530 nm. Krebs-Henseleit solution was used as a blank probe (28).

### Statistical Analysis

Statistical analysis was performed using SPSS 22.0. All results are expressed as mean ± SEM. Statistical significance was determined by the Mann-Whitney *U*-test, and, where appropriate, the Student's independent *t*-test. A *P* < 0.05 was considered statistically significant.

## Results

### Expression of Galectin 3 in Islets and Serum Levels of Galectin 3 Are Increased in Mice on a High-Fat Diet

**Figure 1:**
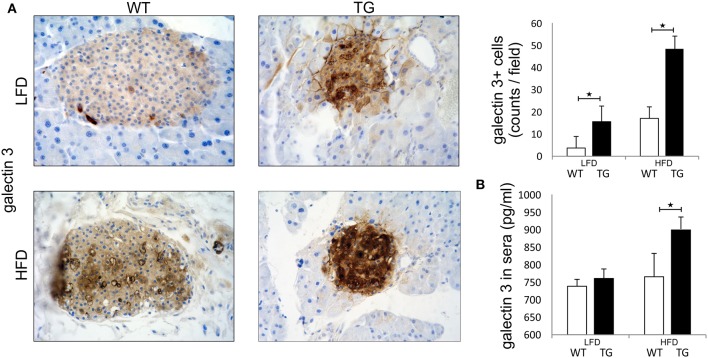
Expression of galectin 3 is increased in mice on a high-fat diet. Immunohistochemical expression of galectin 3^+^ cells in islets. The number of galectin 3 immunopositive cells was significantly higher in TG mice compared to WT mice on standard diet. On HFD, the number of galectin 3 immunopositive cells in islets increased in both groups and was significantly higher in TG mice compared to WT **(A)**. Levels of galectin 3 in sera. By week 16 of HFD, levels of galectin 3 in sera were also significantly higher in TG mice compared to WT mice **(B)**. An analysis was performed by light microscope using a 40X magnifying lens. Results are presented as a mean count of positive cells per field on 10 non-overlapping fields/section. Data from two experiments with 10–15 mice per group are shown as mean ± SEM; by Mann-Whitney *U*-test and independent-sample Student's *t*-test; *p < 0.05.

Pathohistological analysis revealed that the number of galectin 3 immunopositive cells was significantly higher in TG mice compared to WT mice on standard diet. On HFD, the number of galectin 3 cells in islets increased in both WT and TG mice. However, there was a significant difference between the number of galectin 3 positive cells in WT and TG mice on HFD (Figure 1A). By week 16 of HFD, levels of galectin 3 in sera were also significantly higher in TG mice compared to WT mice. Under HFD conditions, both experimental groups of mice had an increase in serum and tissue gal-3, but this increase was significantly higher in the TG mouse group. We find that, under HFD conditions, this increase is positively correlated with the length of treatment and the initial gal-3 concentration (Figure 1B).

### Overexpression of Galectin 3 in β Cells Leads to Impaired Glucose Tolerance and Insulin Resistance (IR) in Mice on a High-Fat Diet

**Figure 2:**
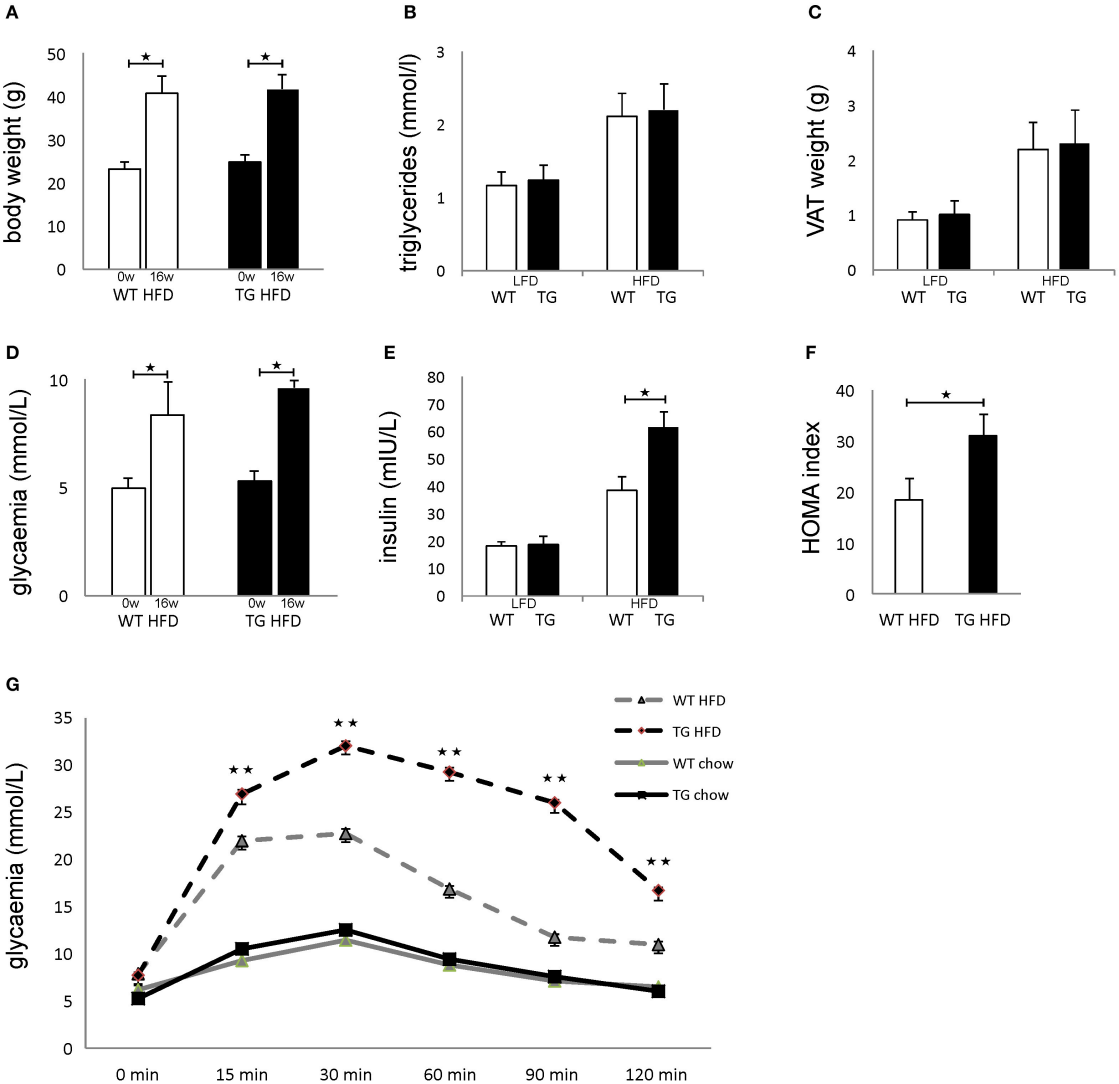
Overexpression of galectin 3 in islets leads to the development of impaired glucose tolerance and insulin resistance *in vivo*. Body weight at the beginning and after 16 weeks on an HFD regime. Both WT and TG mice showed increased body weight after 16 weeks of HFD **(A)**. Levels of triglycerides in sera after 16 weeks of LFD and HFD feeding. There was no difference in triglyceride levels in sera between the two groups **(B)**. Weight of epididymal VAT after 16 weeks on LFD and HFD. There was no difference in the weight of epididymal fat deposits between the two groups **(C)**. Blood glucose levels after 4 h of fasting at the beginning and after 16 weeks on HFD feeding. After 16 weeks of HFD, both WT and TG mice showed increased glycemia after 4 h of fasting, but there was no difference between the two groups **(D)**. Serum insulin levels after 16 weeks and HOMA index. Insulin levels in mice on HFD were significantly higher in TG mice compared to WT mice. The HOMA index was also significantly higher in TG mice on HFD **(E,F)**. Glucose tolerance test after 16 weeks. Glycaemia at 15, 30, 60, 90, and 120 min after glucose application was significantly higher in TG mice on HFD **(G)**. Blood glucose levels after 12 h of fasting. After 16 weeks of HFD, both WT and TG mice showed increased glycemia after 12 h of fasting, but there was no difference between the two groups **(D)**. Data from two experiments with 10–15 mice per group are shown as mean ± SEM; by Mann-Whitney *U*-test and independent-sample Student's *t*-test; *p < 0.05, **p < 0.01.

Both WT and TG mice showed increased body weight after 16 weeks of HFD in comparison with mice on standard diet, but there was no difference between the two groups of animals (Figure 2A). There was no difference in triglycerides in sera and weight of epididymal fat deposits between the two groups (Figures 2B,C). After 16 weeks of HFD, both WT and TG mice showed increased glycemia in comparison with mice on standard diet, but there was no difference between the two groups of animals (Figure 2D). In contrast, insulin levels in mice on HFD were significantly higher in TG mice (Figure 2E). Therefore, the HOMA index was also significantly higher in TG mice on HFD (Figure 2F). Despite the fact that there were no significant differences in fasting glycemia, the glucose tolerance test revealed that glycemia at 15, 30, 60, 90, and 120 min after glucose application was significantly higher in TG mice (Figure 2G).

### Transgenically Enhanced Galectin 3 Expression on β Cells Aggravates Islet Inflammation in Mice on HFD

**Figure 3:**
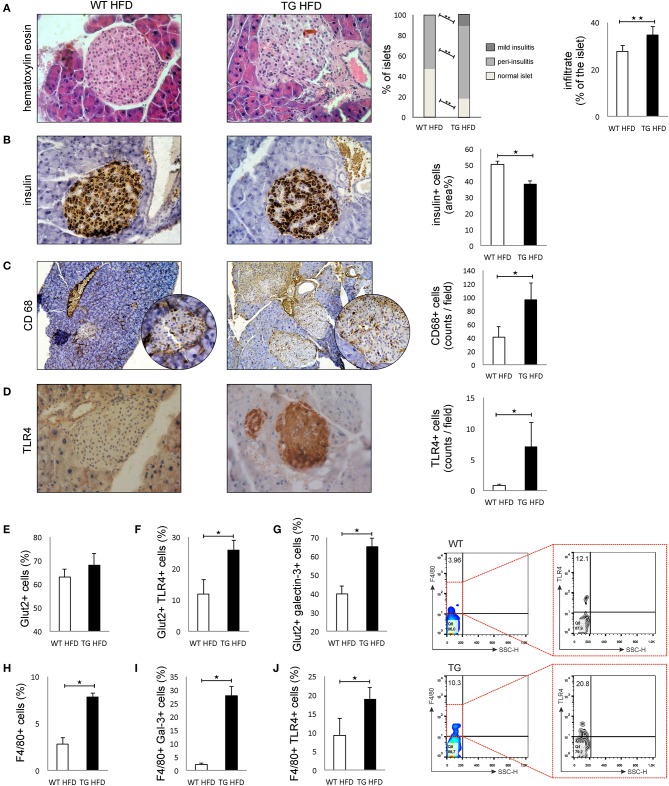
Transgenically enhanced galectin 3 expression on β cells aggravates islet inflammation and increases TLR4 expression. Islet infiltration after 16 weeks of HFD feeding. Percentage of islets with peri-insulitis and mild insulitis was higher in TG mice, and the intensity of mononuclear cell infiltration in the islets was significantly higher in TG mice compared to WT **(A)**. Immunohistochemical expression of insulin+ cells in islets. The percentage of insulin-positive islet area was significantly lower in TG mice **(B)**. Immunohistochemical expression of CD68^+^ cells in islets. A significantly higher number of CD68^+^ macrophages was observed in TG mice on HFD compared to WT mice **(C)**. Immunohistochemical expression of TLR4^+^ cells in islets. A significantly higher number of TLR4^+^ cells was observed in TG mice on HFD compared to WT mice **(D)**. Percentage of β cells, TLR4^+^ and galectin 3-positive β cells in islets. Analysis of the islet cells did not show a significant difference in the total number of β cells in islets in the two experimental groups on HFD. However, there was a significantly higher percentage of Glut-2-positive cells expressing TLR4 and galectin 3 in the TG mouse group **(E–G)**. Percentages of F4/80^+^, F4/80^+^TLR4^+^, and F4/80^+^ Galectin3^+^ cells in islets. Representative contour plots for F4/80^+^TLR4^+^ cells in islets. The percentage of F4/80^+^ macrophages, as well as the percentage of galectin 3-positive and TLR4-positive macrophages, was significantly higher in the islets of TG mice **(H–J)**. An analysis was performed by light microscope using a 40X magnifying lens. Results are presented as a mean count of positive cells per field or percentage of islet area on 10 non-overlapping fields/section. Data from two experiments with 10–15 mice per group are shown as mean ± SEM; by Mann-Whitney *U*-test and independent-sample Student's *t*-test; *p < 0.05, **p < 0.01.

Patohistological analysis revealed that the percentage of islets with peri-insulitis and mild insulitis was higher in TG mice (Figure 3A). The intensity of mononuclear cell infiltration in the islets was significantly higher in TG mice compared to WT (Figure 3A). Further, the percentage of insulin-positive islets area was significantly lower in TG mice (Figure 3B).

Immunohistochemical analysis of pancreatic islets showed a significantly higher number of CD68^+^ macrophages in TG mice on HFD compared to WT mice (Figure 3C). Similarly, expression of TLR4 was significantly higher in the islets of TG mice (Figure 3D). Phenotypic analysis of the islet cells did not show a significant difference in the total number of β cells in islets in the two experimental groups on HFD (Figure 3E). However, there was a significantly higher percentage of TLR4- and galectin 3-positive cells in the group of mice with transgenically enhanced galectin 3 expression (Figures 3F,G). The percentage of F4/80^+^ macrophages, as well as a percentage of galectin 3-positive and TLR4-positive macrophages, was significantly higher in the islets of TG mice (Figures 3H–J).

### Overexpression of Galectin 3 Increases β-Cell Apoptosis in the HFD Condition *in vivo* and After Stimulation With Cytokines and Palmitic Acid *in vitro*

**Figure 4:**
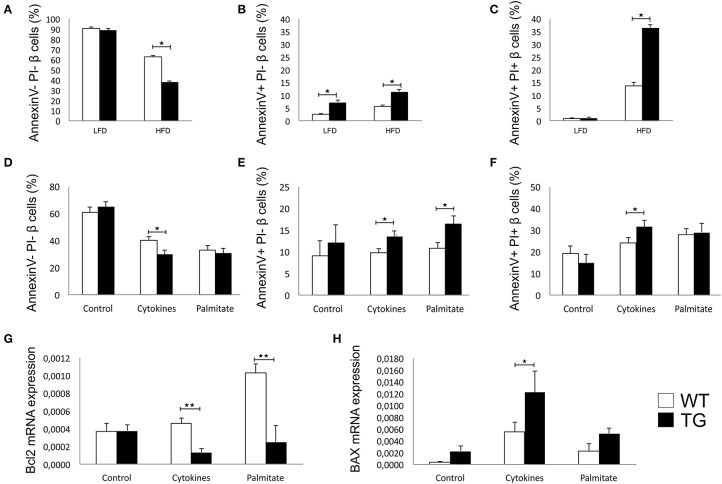
Galectin 3 overexpression increases β-cell apoptosis in HFD conditions *in vivo* and after cytokine or palmitic acid stimuli *in vitro*. Percentage of viable β cells (Annexin V^−^ PI^−^) after 16 weeks of LFD and HFD feeding. There was no difference in the percentage of viable β cells between WT and TG mice on LFD, while in TG mice on HFD, we found a significantly lower percentage of healthy β cells **(A)**. Percentage of early apoptotic β cells (Annexin V^+^ PI^−^) after 16 weeks of LFD and HFD feeding. The percentage of early apoptotic cells was significantly higher in TG mice compared to WT on both diets **(B)**. Percentage of late apoptotic β cells (Annexin V^+^ PI^+^) after 16 weeks of LFD and HFD feeding. The percentage of late apoptotic β cells was significantly higher in TG mice on HFD compared to WT mice **(C)**. Percentage of viable β cells (Annexin V^−^ PI^−^), early apoptotic β cells (Annexin V^+^ PI^−^), and late apoptotic β cells (Annexin V^+^ PI^+^) *in vitro*. Analysis of isolated pancreatic islets demonstrated that there was no difference between groups in the percentage of viable or apoptotic unstimulated β cells, but after 24-h stimulation with proinflammatory cytokine cocktail (IFN-γ, IL-1β, and TNF-α), the percentage of viable cells was significantly lower while the percentage of early and late apoptotic β cells was significantly higher in TG mice. Also, stimulation with palmitic acid significantly increased the percentage of early apoptotic cells in TG mice islets **(D–F)**. Relative expression of Bcl2 and BAX mRNA *in vitro*. Real time RT-PCR analysis revealed no differences in basal levels of anti-apoptotic Bcl2 mRNA between the two groups of islet cells. However, basal expression of pro-apoptotic BAX was significantly higher in islets of TG mice. After 24-h stimulation with proinflammatory cytokines or palmitic acid, the expression of Bcl2 was significantly lower in islets with overexpressed galectin 3, while the expression of BAX was increased in both groups and significantly higher in islets of TG mice **(G,H)**. Data from two experiments with 5–8 mice per group are shown as mean ± SEM; by Mann-Whitney *U*-test and independent-sample Student's *t*-test; *p < 0.05, **p < 0.01.

There was no difference between the percentages of viable β cells in WT and TG mice on LFD, while in TG mice on HFD, we found a significantly lower percentage of healthy β cells (Figure 4A). The percentage of early apoptotic cells was significantly higher in TG mice compared to WT on both diets (Figure 4B). Also, the percentage of late apoptotic β cells was significantly higher in TG mice on HFD, as evaluated by annexin V and PJ staining (Figure 4C).

We isolated pancreatic islets from TG and WT animals on a chow diet to evaluate inherent susceptibility to apoptosis. There was no difference between groups in the percentage of viable or apoptotic unstimulated β cells, but after 24 h of stimulation with a proinflammatory cytokine cocktail (IFN-γ, IL-1β, and TNF-α), the percentage of viable cells was significantly lower, while the percentage of early and late apoptotic β cells was significantly higher in TG mice (Figures 4D–F). Stimulation with palmitic acid significantly increased the percentage of early apoptotic cells in TG mice islets (Figure 4E).

Real time RT-PCR revealed no differences in basal levels of anti-apoptotic Bcl2 mRNA between the two groups of islet cells (Figure 4G). However, basal expression of pro-apoptotic BAX was significantly higher in islets of TG mice (Figure 4H). After 24 h of stimulation with proinflammatory cytokines or palmitic acid, the expression of Bcl2 was significantly lower in islets with overexpressed galectin 3, while the expression of BAX was increased in both groups and was significantly higher in islets of TG mice (Figures 4G,H).

### Galectin 3-Overexpression Increases the Oxidative Stress of β Cells *in vitro*

**Figure 5:**
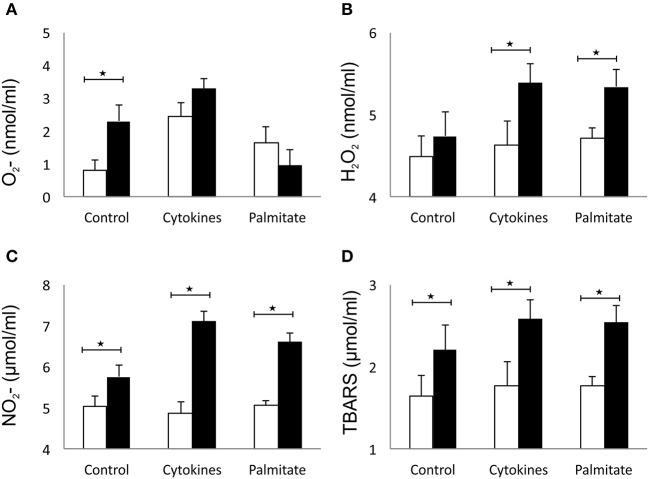
Galectin 3 overexpression increases the oxidative stress of β cells *in vitro*. Overexpression of galectin 3 led to an inherent capacity of islet cells to produce oxygen free radical O_2−_ in the control group, while there were no differences after stimulation with an IFN-γ, IL-1β, TNF-α cocktail **(A)**. Overexpression of galectin 3 exerted a prooxidant effect and increased levels of H_2_O_2_ after 24-h stimulation with proinflammatory cytokine cocktail **(B)**. Galectin 3 overexpression plays a prooxidant role independently of cytokine stimuli, as evaluated by levels of NO and thiobarbituric acid reactive substances (TBARS) **(C,D)**. Data from two experiments with 5–8 mice per group are shown as mean ± SEM; by Mann-Whitney *U*-test and independent-sample Student's *t*-test; *p < 0.05.

The effect of overexpressed galectin 3 on the parameters of oxidative stress in β cells was analyzed by measuring levels of O_2−_, H_2_O_2_, NO, and TBARS in 24-h supernatants of islet cells. Overexpression of galectin 3 led to an inherent capacity of islet cells to produce oxygen free radical O_2−_ in the control group, while there were no differences after stimulation with the IFN-γ, IL-1β, TNF-α cocktail (Figure 5A). Overexpression of galectin 3 exerted a prooxidant role and increased levels of H_2_O_2_ after 24-h stimulation with proinflammatory cytokine cocktail (Figure 5B). Also, galectin 3-overexpression has a prooxidant effect independently of cytokine stimuli, as evaluated by levels of NO and thiobarbituric acid reactive substances (TBARS) (Figures 5C,D).

### Increased Islet Inflammation in Mice With Enhanced Galectin 3 Expression on β Cells Is Accompanied by a Macrophage Shift Toward a Proinflammatory Phenotype in Pancreatic Lymph Nodes

**Figure 6:**
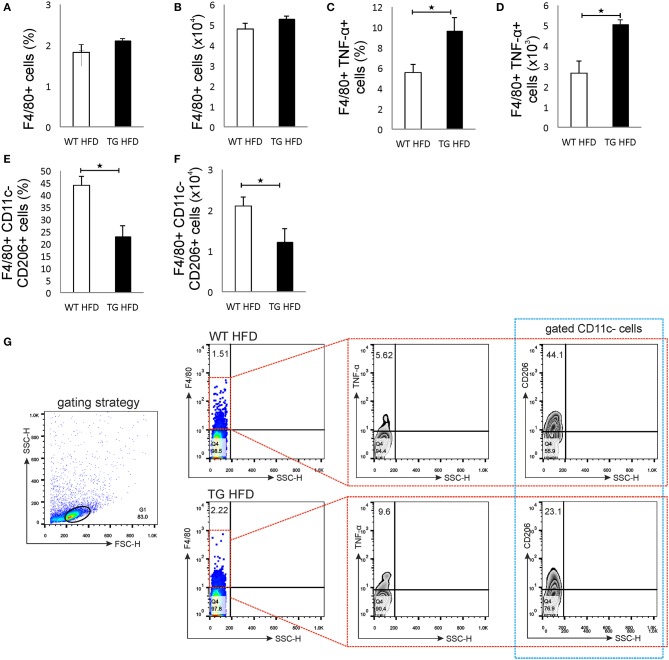
Islet inflammation is accompanied by an enhanced number of F4/80^+^TNF-α^+^ M1 macrophages and a decreased number of F4/80^+^CD206^+^ M2 macrophages in pancreatic lymph nodes. Percentages and total numbers of F4/80^+^ macrophages in pancreatic lymph nodes. There were no differences in the percentage and number of F4/80^+^ macrophages in mice on HFD **(A,B)**. Percentages and total numbers of F4/80^+^TNF-α^+^ M1 macrophages. Percentage and number of proinflammatory M1 F4/80^+^TNF-α^+^ macrophages were significantly higher in pancreatic lymph nodes of TG mice on HFD compared to WT **(C,D)**. Percentages and total numbers of F4/80^+^CD11c^−^CD206^+^ M2 macrophages. The percentage and number of M2 F4/80^+^CD11c^−^CD206^+^ macrophages were significantly lower in pancreatic lymph nodes of TG mice on HFD **(E,F)**. Gating strategy, representative dot and contour plots for F4/80^+^, F4/80^+^TNF-α^+^, and F4/80^+^CD11c^−^CD206^+^ cells in pancreatic lymph nodes **(G)**. Data from two experiments with 10–15 mice per group are shown as mean ± SEM; by Mann-Whitney *U*-test and independent-sample Student's *t*-test; *p < 0.05.

Phenotypic analysis of the pancreatic lymph nodes demonstrated that there were no differences in the percentage and number of F4/80^+^ macrophages in mice on HFD (Figures 6A,B,G). However, the percentage and number of proinflammatory M1 F4/80^+^TNF-α^+^ macrophages were significantly higher in pancreatic lymph nodes of TG mice on HFD compared to WT (Figures 6C,D,G). Further, the percentage and number of M2 F4/80^+^CD11c-CD206^+^ macrophages were significantly lower in pancreatic lymph nodes of TG mice on HFD (Figures 6E,F,G).

## Discussion

Obesity leads to low-grade chronic inflammation in metabolic tissues that is not only associated with insulin resistance but is also important for β cell function (10). Galectin 3 appears to be important for the development of insulin resistance in target tissues. Li et al. elegantly demonstrated that exogenous administration of galectin 3 causes insulin resistance and glucose intolerance in mice (29).

To study the effect of galectin 3 expression on β cells on their function in obesity and type-2 diabetes, we combined *in vivo* and *in vitro* approaches. Histological and immunohistological analysis showed that galectin 3 overexpression facilitates β-cell damage as evaluated by the size of the insulin-positive area of the islets (Figure 3) and, in particular, β-cell apoptosis (Figure 4).

Enhanced β-cell damage in obesity was correlated with an increased percentage of inflammatory macrophages expressing galectin 3 and TLR4 in the islets of TG mice (Figure 3). Our study did not determine whether the increased number of M1 macrophages is due to the influx of blood-borne cells or due to the shift of resident macrophages toward M1 phenotype. Previous studies suggested that in obesity, type-2 diabetic islets express higher levels of chemokines, which leads to increased macrophage content due to the recruitment of bone-marrow-derived blood monocytes (30, 31). Cucak et al. demonstrated that M1-like galectin 3^+^ macrophages invaded diabetic islets (32). On the other hand, Ying et al. recently demonstrated that obesity induces the local expansion of resident intra-islet macrophages, independent of the recruitment of circulating monocytes (33).

The activation of the TLR4 receptor by saturated NEFA in HFD-fed mice might activate the inflammasome in β cells and lead to their damage (30, 34–38). This may be an additional mechanism that leads to further damage and reduction of β cells. An increased amount of galectin 3 in islets and an increased production of pro-inflammatory cytokines leads to the development of IR that is more pronounced in the group of mice with transgenically enhanced galectin 3 expression. This leads to increased β-cell stress, and their damage may additionally reduce the number of β cells. Recent studies suggested that galectin 3 acts as an endogenous TLR-4 ligand and promotes neuroinflammation (39). We demonstrated increased expression of TLR-4 and galectin 3 on β cells and F4/80^+^ macrophages in islets, which is associated with aggravated islets inflammation in TG mice (Figure 3). This result, implicating that galectin 3 acts as a ligand for TLR-4, might also be important in the pathogenesis of type-2 diabetes.

**Figure 7:**
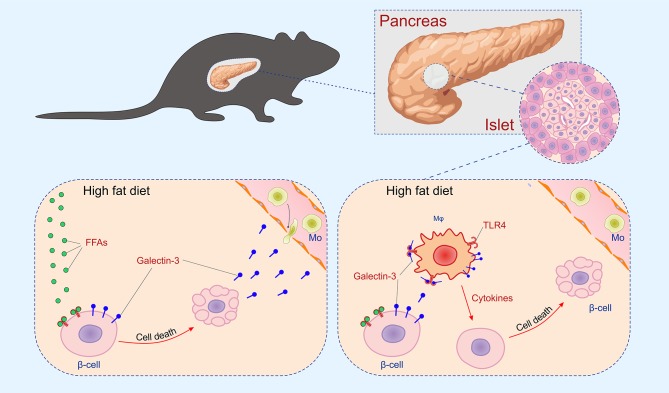
Mechanism of islet inflammation aggravated by over-expression of galectin 3 on β cells. Saturated NEFA, increased by HFD feeding, activate TLR4 on β cells and induce their initial damage **(Left)**. Galectin 3 released from damaged β cells in a paracrine manner stimulates macrophage migration to the islet, which aggravates inflammation and further cell damage **(Right)**.

The initial damage of β cells overexpressing galectin 3 as a result of HFD led to increased serum galectin 3 (Figure 1B). It has been shown that circulating galectin 3 has a proinflammatory effect, which correlates with our findings (40). Galectin 3 released from β cells due to initial damage, acts as alarmin and paracrine chemotactic factor for inflammatory cells, promoting macrophage migration and activation in the islets, leading to enhanced islet inflammation and apoptosis (31, 41, 42). The proposed mechanism is shown in Figure 7. The alterations within the islets are reflected in the cellular makeup of the draining lymph node and the clinical and histological parameters of type-2 diabetes. Phenotypic analysis of the cells in pancreatic lymph nodes demonstrated that the percentage and number of proinflammatory M1 F4/80^+^TNF-α^+^ macrophages were significantly higher in pancreatic lymph nodes of TG mice on HFD compared to WT (Figures 6C,D). Further, the percentage and number of M2 F4/80^+^CD11c^−^CD206^+^ macrophages were significantly lower in pancreatic lymph nodes of TG mice on HFD (Figures 6E,F).

In mice with transgenically overexpressed galectin 3, fasting glycemia was similar to the WT control group on HFD but with significantly higher compensatory hyperinsulinemia as a consequence of increased activity and insulin production by β cells. The presence of insulin resistance with compensatory hyperinsulinemia was additionally burdened by the reduced number of healthy β cells. In such circumstances, due to increased insulin requirements, β cells increase the production of reactive oxygen species (ROS), which leads to apoptosis, which additionally reduces the number of remaining healthy β cells compared to the control mice on HFD (43, 44). Compensatory hyperinsulinemia and increased insulin production, in mice with transgenically enhanced galectin 3 expression, is enough to sustain relatively normal fasting glycemia compared to the control group on HFD but is insufficient after glucose load (Figure 1). Recent studies have clearly shown that soluble galectin 3 strongly influences the development of insulin resistance (29). The state of insulin resistance in the fasting state, in our case, can be estimated using fasting glycemic values (after 4 h without food), fasting insulinemia (after 4 h without food), the insulin resistance index, and serum solubility galectin 3. All of these parameters were significantly higher in the experimental groups on HFD, and most of these parameters were significantly higher in the transgenic group of mice compared to the control group on the same diet. This clearly indicates a significant degree of insulin resistance in a transgenic mouse group at the fasting period (45). Significantly increased glycemia after an overnight fasting period of 12 h, especially in the experimental groups on HFD, clearly supports the existence of hepatic insulin resistance and a failure of insulin to suppress hepatic glucose production overnight, which is in line with our results (46). On the other side, disruption of glucose homeostasis after glucose loading can be assessed by glycemic values during ipGTT. In the condition of fasting insulin resistence, after glucose loading, the presence of marked hyperglycemia during ipGTT can be interpreted as a further worsening of resistance and/or reduced production of insulin. The most severe disorder of glucose homeostasis was seen in a group of transgenic mice on HFD. All these results, taken together, make it quite clear that there is a significant degree of insulin resistance in the HFD group and especially in the TG mouse group, which leads to a more severe form of glucose metabolism disorder. As is already known, insulin treatment is very important but does not represent the first steps in treating this condition. Since the pathogenesis of diabetes is multifactorial, the initial steps in treatment would be to improve insulin sensitivity in the liver and peripheral organs, to reduce glucagon production, to increase insulin production from the remaining β cells, or to stimulate glycosuria, while basal insulin use would be the initial step in insulin therapy (47). We are fully aware that our results presented here do not support those obtained by Pejnovic et al. (48). Additional experiments demonstrated that the WT mice in our Diabetes paper were not fully adequate and showed unexpectedly low glycemia on a high-fat diet. This is in agreement with the paper by Li et al. (29). In the experiments in this paper, we used littermate controls and transgenic mice, all obtained from the laboratory of Prof. Bernard Thorens, University of Lausanne.

Glycotoxicity, which occurs as a result of more pronounced post-prandial hyperglycemia, is more expressed in the group of mice with transgenically enhanced galectin 3 expression, which further leads to β-cell damage and reduces the number of remaining healthy β cells compared to WT control mice on HFD (49–51).

There is ample evidence that galectin 3 plays a role in the regulation of apoptosis (52, 53). Depending on the cell type and cell stimuli, galectin 3 may have anti- or pro-apoptotic activity (53, 54). Saksida et al. showed that genetic deletion of galectin 3 in β cells enhanced the resistance to cytokine-triggered apoptosis and, in particular, affects the components of the mitochondrial apoptotic pathway (20). We demonstrated that galectin 3 overexpression increased the percentage of apoptotic islet cells *in vivo* (Figure 4). Also, in islet cells stimulated with cytokines and palmitic acid, galectin 3 overexpression enhances the expression of pro-apoptotic BAX and apoptosis (Figure 4). Our findings are in line with previous results (20) that showed that deletion or inhibition of galectin 3 promotes survival and preserves β-cell function. The overexpression of galectin 3 on β cells also increases apoptosis, whether induced by cytokines or NEFA (Figure 4). It appears that apoptosis of β cells in TG islets leads to an increased level of circulating galectin 3, leading to systemic alteration of glycoregulation. This confirms that islet cells are active participants in the development of type-2 diabetes.

β-cell death is clearly dependent on oxidative stress and free radicals. Early studies implicated nitric oxide as the effector molecule responsible for the detrimental effects of pro-inflammatory cytokines on β-cell function (55, 56). The role of nitric oxide in β-cell pathophysiology has been investigated over the years, and recent findings suggest its dual role as a molecule that inhibits insulin secretion but also as an activator of protective pathways, which protects β cells from damage (57, 58). Our results demonstrated that overexpression of galectin 3 is related to increased levels of nitrite oxide, which is followed by its deleterious effects on β cells (Figure 5).

In conclusion, we provide direct evidence that expression of galectin 3 facilitates β-cell damage induced by cytokines and NEFA, therefore promoting type-2 diabetes. These results and previous findings that galectin 3 on invading immune cells promotes β-cell damage *in vivo* indicate that galectin 3 might be a therapeutic target in type-2 diabetes.

## Data Availability Statement

The datasets generated for this study are available on request to the corresponding author.

## Ethics Statement

This animal study was reviewed and approved by Ethical Committee of the Faculty of Medical Sciences, University of Kragujevac (Permit Number: 01-6675).

## Author Contributions

IP, NP, BL, SP, MM, ND, IJ, MA, and NJ performed experiments, did the statistical analyses, and participated in data interpretation. IP, NJ, ND, AD, MA, and MM analyzed data, did the statistical analyses, and participated in data interpretation. ML, NP, and NJ conceived and designed experiments. ML, NA, NP, IP, and NJ wrote the article. All authors gave final approval of the version to be published and are responsible for the integrity of the work as a whole.

### Conflict of Interest

The authors declare that the research was conducted in the absence of any commercial or financial relationships that could be construed as a potential conflict of interest.
