# Supplementary material for: Overexpression of Galectin 3 in Pancreatic β Cells Amplifies β-Cell Apoptosis and Islet Inflammation in Type-2 Diabetes in Mice
Source: Front Endocrinol (Lausanne). 2020 Feb 7;11:30. doi: 10.3389/fendo.2020.00030 (PMC7018709; doi:10.3389/fendo.2020.00030)
Supplement: Supplementary file 1 [file Data_Sheet_1.docx]

**Supplement 1.**

| Primers (Kapa Biosystems, USA) | |
| --- | --- |
| Forward | ATGCTCAGCCAAGGACAAAG (On RIP Promoter) |
| Reverse | AGTTGGGCCAGGATAAGCTC (On Lgals3 cDNA) |

Supplement 1: PCR confirmation of genotype. PCR reaction was performed using a specific set of primers (Kapa Biosystems, USA), and the presence of a 591bp product was visualized on agarose gel. In this example (picture 1), 591bp product (tg/+ LGALS3 OE) was present in four specimens (5,7,9,11).
