# Supplementary material for: Overexpression of Galectin 3 in Pancreatic β Cells Amplifies β-Cell Apoptosis and Islet Inflammation in Type-2 Diabetes in Mice
Source: Front Endocrinol (Lausanne). 2020 Feb 7;11:30. doi: 10.3389/fendo.2020.00030 (PMC7018709; doi:10.3389/fendo.2020.00030)
Supplement: Supplementary file 2 [file Data_Sheet_2.docx]

**Supplement 2.**

**
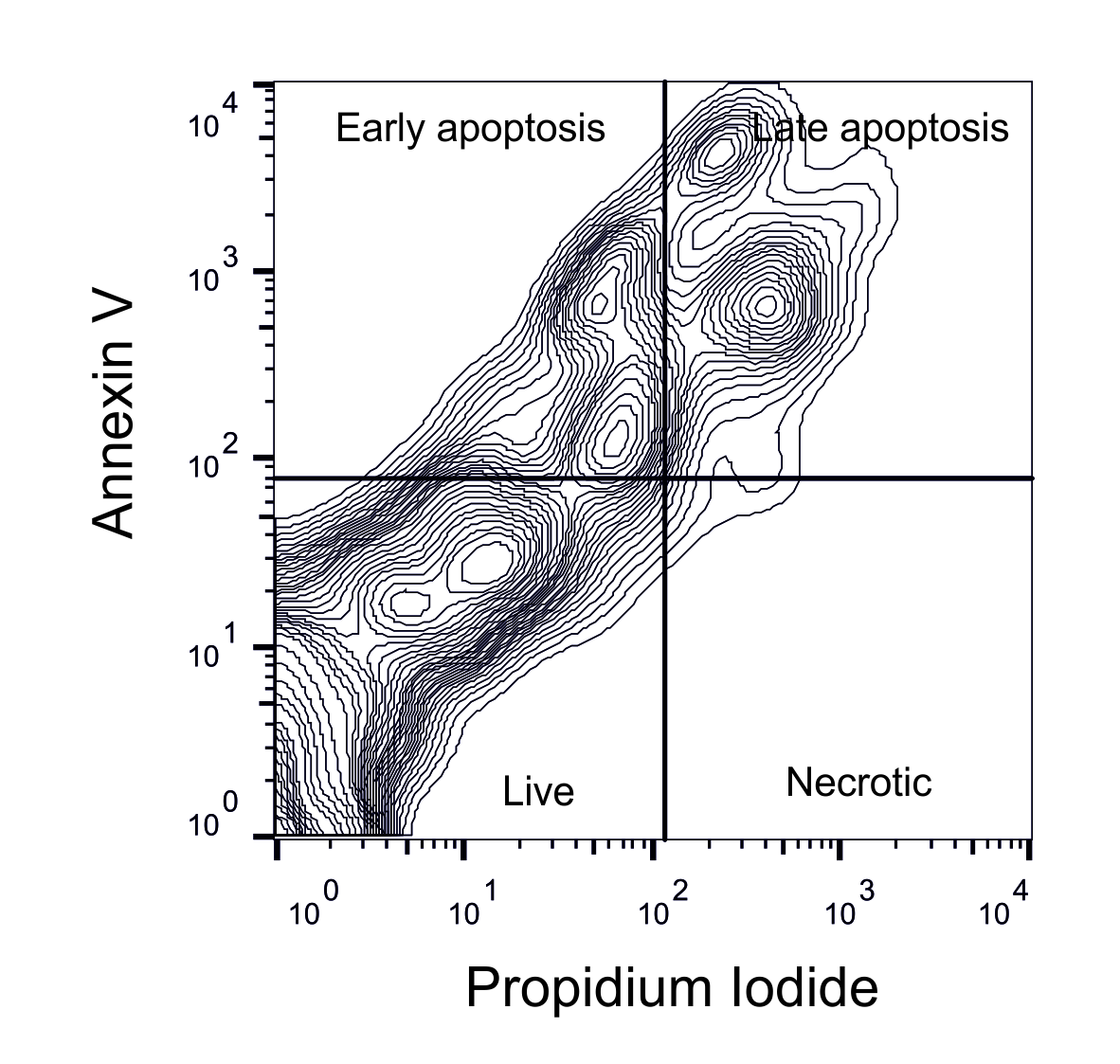
**

Supplement 2: Contour plot representing the analysis strategy for assessment of cell apoptosis.
